# Acceleration of skeletal maturation in Central Europe over the last two decades: insights from two cohorts of healthy children

**DOI:** 10.1007/s00247-024-05994-6

**Published:** 2024-07-20

**Authors:** Johanna Pape, Maciej Rosolowski, Peter Zimmermann, Roland Pfäffle, Franz W. Hirsch, Daniel Gräfe

**Affiliations:** 1 Department of Pediatric Radiology, University Hospital Leipzig, Liebigstraße 20 a, 04103 Leipzig, Germany; 2 Institute for Medical Informatics, Statistics and Epidemiology, Leipzig University, Leipzig, Germany; 3 Department of Pediatric Surgery, University Hospital, Leipzig, Germany; 4 Department of Pediatrics, University Hospital, Leipzig, Germany

**Keywords:** Artificial intelligence, Bone age, Children growth disorders, Precocious puberty, Reference growth curves

## Abstract

**Background:**

Deviations between the determination of bone age (BA) according to Greulich and Pyle (G&P) and chronological age (CA) are common in Caucasians. Assessing these discrepancies in a population over time requires analysis of large samples and low intra-observer variability in BA estimation, both can be achieved with artificial intelligence-based software. The latest software-based reference curve contrasting the BA determined by G&P to the CA of Central European children dates back over two decades.

**Objective:**

To examine whether the reference curve from a historical cohort from the Netherlands (Rotterdam cohort) between BA determined by G&P and CA still applies to a current Central European cohort and derive a current reference curve.

**Materials and methods:**

This retrospective single-center study included 1,653 children and adolescents (aged 3–17 years) who had received a radiograph of the hand following trauma. The G&P BA estimated using artificial intelligence-based software was contrasted with the CA, and the deviations were compared with the Rotterdam cohort.

**Results:**

Among the participants, the mean absolute error between BA and CA was 0.92 years for girls and 0.97 years for boys. For the ages of 8 years (boys) and 11 years (girls) and upward, the mean deviation was significantly greater in the current cohort than in the Rotterdam cohort. The reference curves of both cohorts also differed significantly from each other (*P* < 0.001 for both boys and girls).

**Conclusion:**

The BA of the current Central European population and that of the curve from the Rotterdam cohort from over two decades ago differ. Whether this effect can be attributed to accelerated bone maturation needs further evaluation.

## Introduction

Determination of bone age (BA) is important for diagnosing and assessing the progression of various childhood and adolescent diseases. BA is most frequently assessed using the atlas of Greulich and Pyle (G&P), which employs data gathered from children more than 80 years ago. The atlas was published in 1959, based on an analysis of the hand radiographs of 1,000 upper-class children in Ohio between 1931 and 1942. For each chronological year of age and sex, a representative radiograph was used as a reference [1].

For many years, the rating was used almost exclusively by radiologists and pediatricians, who employed the printed version of the G&P atlas. With the introduction of the software BoneXpert in 2008 (Visiana, Hørsholm, Denmark), which was approved for the European market with the “conformité européenne” (CE) certificate, automated estimation of BA has become widespread over the last 15 years [2]. Since then, different studies have proven that the accuracy of the program reaches the accuracy level of an expert in BA determination.

However, socioeconomic changes and the modifications they could have caused in skeletal maturity over the last 100 years raise doubts about whether the G&P atlas corresponds to the chronological age (CA) of today’s Caucasian children [3–5]. The deviation between BA determined by the G&P atlas and CA was analyzed 15 years ago, when a study by van Rijn et al. determined BA in a collective of 405 healthy Dutch children in 1997 using BoneXpert [2]. In this study, the researchers found only minor deviations between BA, as estimated by BoneXpert, and CA and created a reference curve with standard deviations (SD) (referred to below as the Rotterdam reference curve). For radiation protection and ethical reasons, approval for an X-ray series of healthy children in Central Europe is very difficult to obtain today, and the results of van Rijn et al.’s study have yet to be reproduced. However, hand radiographs of children performed following trauma offer an alternative. A large sample size should allow individual outliers with growth disorders to be identified in this collective.

The current study aims to examine whether the Rotterdam reference curve between BA determined by G&P and CA still applies to a current Central European cohort and derive a current reference curve.

## Material and methods

### Contemporary cohort

For this retrospective study, a population of 1,653 patients already described in a previous study was employed [6]. In summary, the cohort comprised patients between 1 year and 18 years of age who had received a radiograph of the left or right hand following trauma between 2012 and 2022. We restricted the age range of the study to 3–17 years for boys and 3–15 years for girls.

The motivation for the lower limit of 3 years was that the Leipziger collective data lacked statistical power at lower ages. The motivation for the upper limit of 17 years for boys was that the BA scale in BoneXpert ends at a BA of 19 years. Thus, the results of adolescents reaching that age would be skewed towards lower BA. Finally, the upper limit of 15 years for girls was chosen to maintain consistency with the limit of 17 years for boys, as a BA of 17 years for boys appears the same as a BA of 15 years for girls.

All images were obtained at a single institute for pediatric radiology (University Hospital, Leipzig, a tertiary care center) using an Axiom Aristos FX machine (Siemens Healthineers, Erlangen, Germany) without a scatter grid and while employing a 0.1-mm copper filter. The left hand or, if exclusively available, the right hand including the wrist was examined in posterior-anterior projection [7]. Two pediatric radiologists (G.D. and J.P., with 15 and 4 years of experience in pediatric radiology, respectively) eliminated images with fractures and images of inadequate quality or positioning in advance. Ethics approval for the retrospective study was obtained from the local ethics committee, and the requirement of informed consent by patients or their legal guardians was waived.

### Historic cohort

The Rotterdam cohort, a historical cohort of Dutch pupils used for reference, is described elsewhere [2]. In summary, the cohort includes 255 Caucasian boys (median age 12.5 years) and 276 Caucasian girls (median age 12.6 years). The radiographs of this Dutch cohort were reanalyzed with the latest version of BoneXpert (3.2.2) at the time of the research, and Visiana kindly provided the tabular results containing mean values and SD between BA and CA for the purposes of the current study.

### Bone age determination

BA was determined automatically in batch processing using BoneXpert 3.2.2 (Visiana) on a local stand-alone server. The results were returned as tabular data and as secondary capture digital imaging and communications in medicine (DICOM) files. Radiographs of low quality (i.e. fewer than eight bones were accepted due to abnormal anatomy or positioning of the hand as well as too sharp or too blurred images) were rejected by BoneXpert [8]. In the contemporary cohort, 34 radiographs (2.0%) were rejected. The radiographs of the contemporary cohort were also evaluated by another artificial intelligence (AI)-based reader, the software PANDA 1.13.21 (ImageBiopsy Labs, Vienna, Austria). This AI-based software is approved as a medical device in Europe for the estimation of BA according to G&P. As no manual G&P determination by human readers was performed for the study, there was neither a “gold standard” for BA, nor was it possible to determine the precision of either AI program.

### Statistics

Statistical analysis was performed using R Studio 2022.07.2 (PBC, Boston, MA). The patients were grouped yearly according to their CA. As the Rotterdam cohort consisted of non-disjoint groups, the age groups in the current collective were formed identically (CA ± 1.5 years). The mean deviation between BA and CA was calculated for each age group. Differences in these mean deviations between the current study cohort and the Rotterdam cohort were examined for each year using Student’s *t*-tests with the Bonferroni correction for multiple testing. To assess whether the reference curves (curves of the mean deviations of BA minus CA across the groups of CA) differed statistically from each other, we performed a global test by aggregating the individual statistics for each age group into one chi-square statistic. This was based on the method of Hristova et al. [9], with a modification for non-disjoint age groups. The modification involved dividing the chi-square statistic and its degrees of freedom by three. This adjustment accounts for the 3-year width of each age group and the 1-year shift between groups [10].

The mean deviation between the BA estimated by BoneXpert and that estimated by PANDA in the cohort from Leipzig was assessed using a paired *t*-test. The deviations between CA and BA were quantified by mean absolute error (MAE) and root mean squared error (RMSE). Characteristics were summarized using medians and interquartile ranges (IQR). The significance level was set at 0.05.

## Results

### Age distribution of the patient cohort

**Fig. 1:**
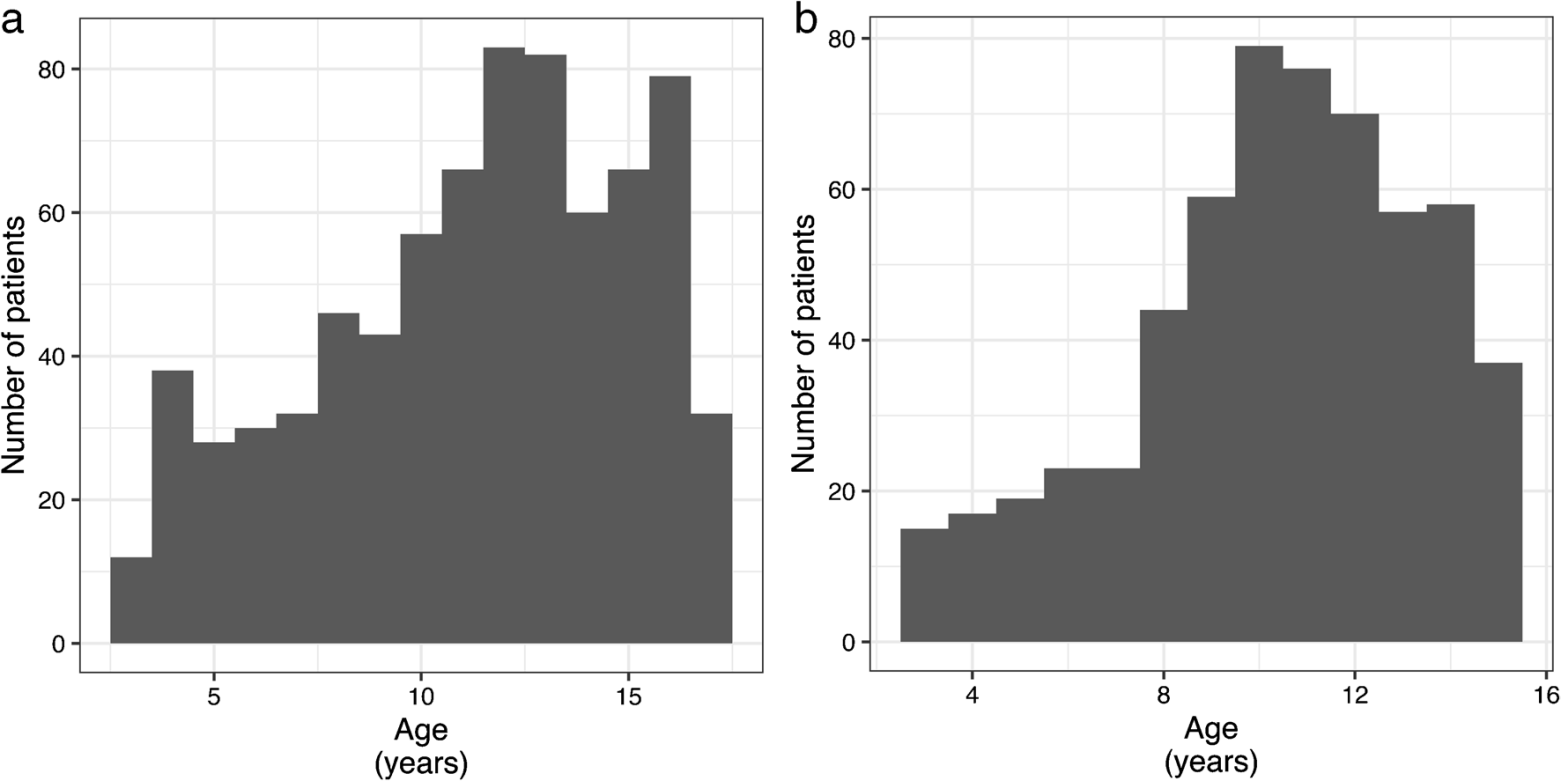
Histogram of age distribution in boys (**a**) and girls (**b**) in the contemporary cohort

A total of 1,331 children (56.6% boys) were included in the retrospective analysis, with a median age of 11.8 years (IQR 8.5–14.3) for boys and 10.7 years (IQR 8.5–12.7) for girls. The age distribution is shown in Fig. 1.

### Analysis of bone age

**Fig. 2:**
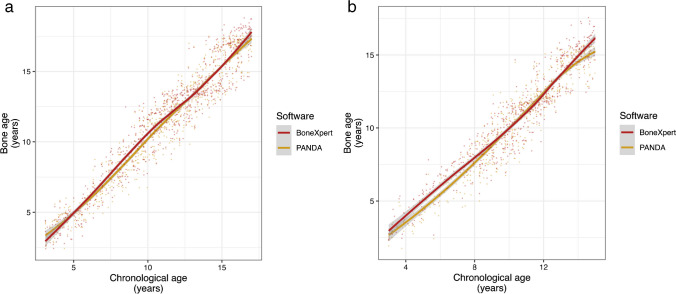
The bone age estimated by BoneXpert and PANDA in relation to chronological age for boys (**a**) and girls (**b**). The solid curve corresponds to the smoothed mean. The mean deviation is very low at 0.14 years (boys) and 0.20 years (girls) (*P* < 0.001)

The MAE between BA and CA in the studied age range was 0.97 years for boys and 0.92 years for girls, and the RMSE was 1.18 years for boys and 1.16 years for girls. The BA estimated by BoneXpert significantly differed from that estimated by PANDA, but only slightly for both boys (mean deviation 0.14 years, *P* < 0.001) and girls (mean deviation 0.20 years, *P* < 0.001) (Fig. 2).

### Analysis of the Rotterdam and the contemporary cohorts

**Fig. 3:**
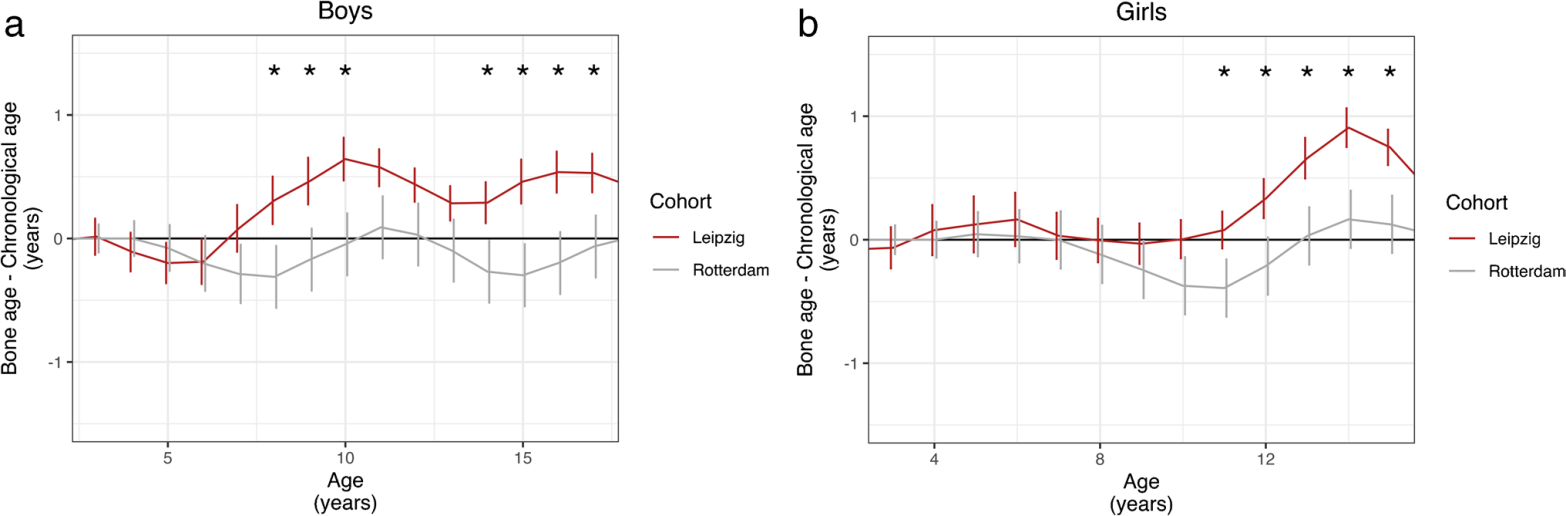
Deviation between Greulich and Pyle bone age and chronological age in children with healthy growth in a historical cohort (Rotterdam cohort) and a contemporary cohort (Leipzig) in boys (**a**) and girls (**b**). Mean values (solid line) and the 95% confidence interval per year of life are shown. The data from the Rotterdam study were kindly provided by van Rijn et al. [2]. * indicates statistically significant age groups (*P* < 0.05)

The mean deviation between G&P BA and CA in the Leipzig collective exceeded that in the Rotterdam cohort in both boys and girls (Fig. 3). This difference was significantly greater in boys of 8 years of age and above and girls of 11 years of age and above (Table 1). In general, the reference curves in both cohorts differed significantly from one another (*P* < 0.001 for boys and girls).

**Table 1:** Mean deviation between bone age and chronological age in the Rotterdam cohort and the Leipzig cohort. The deviations are significantly greater in the Leipzig collective for males from the age of 8 years (with interruptions) and for females from the age of 11 years than in the Rotterdam cohort. The data from the Rotterdam study were kindly provided by van Rijn et al. [2]

Age group	Males			Females		
	Mean Leipzig	Mean Rotterdam	*P*-value	Mean Leipzig	Mean Rotterdam	*P*-value
3	0.01	0.00	1	− 0.07	0.00	1
4	− 0.11	0.00	1	0.08	0.00	1
5	− 0.20	− 0.08	1	0.12	0.04	1
6	− 0.19	− 0.20	1	0.16	0.03	1
7	0.08	− 0.29	0.28	0.03	0.00	1
8	0.31	− 0.31	< 0.01	− 0.01	− 0.12	1
9	0.46	− 0.17	< 0.01	− 0.03	− 0.24	1
10	0.64	− 0.05	< 0.01	0.00	− 0.37	0.12
11	0.57	0.09	< 0.05	0.08	− 0.39	< 0.05
12	0.43	0.03	0.10	0.33	− 0.21	< 0.01
13	0.28	− 0.10	0.15	0.66	0.03	< 0.01
14	0.29	− 0.27	< 0.01	0.91	0.17	< 0.01
15	0.46	− 0.30	< 0.01	0.75	0.12	< 0.01
16	0.54	− 0.20	< 0.01	NA	NA	NA
17	0.53	− 0.06	< 0.01	NA	NA	NA

*NA* not applicable

## Discussion

This study compares the skeletal maturation of a current Central European healthy cohort with that of the Rotterdam cohort, a historical, healthy collective from 25 years ago in a comparable geographical location. One strength of the current study is the large number of cases analyzed, with an average of 83 patients per year of life. Determination of BA by AI not only is economical in terms of personnel resources when dealing with such a high volume of cases but also exhibits a lower precision error than that observed in the case of human readers [2, 11–16]. While the deviation of BA from CA was negligible in younger children, boys under 8 years of age and girls under 11 years of age, a clear acceleration of BA by up to 0.6 years in boys and 0.9 years in girls was observed in older children (boys above 8 years of age and girls above 11 years of age). A misjudgment of BA by the AI software is very unlikely, given its repeatedly documented reliability and high concordance with the estimation of BA by another AI-supported program. Several studies have examined whether the BA according to G&P corresponds to the CA of a current Caucasian population [17]. A meta-analysis in more recent populations (up to a decade ago) found that the G&P atlas, although of limited accuracy in Asian and African populations, is still reliable in Caucasians today [17]. Nevertheless, an acceleration of BA was reported 40 years ago, especially in adolescents during and after puberty, though on a lower level compared to that in our study (0.2 years in boys, 0.13 years in girls) [18]. A more recent study conducted in Germany found larger differences between BA and CA, by 0.49 years in boys and 0.39 years in girls, which is more similar to our results [19]. It appears that the overestimation of BA mainly occurs in older children, whereas BA tends to be underestimated in younger patients [6, 20]. However, in the cohorts examined in the studies mentioned before, no significant deviations were found between BA from CA. It is, therefore, more interesting to note that the Leipzig collective differs significantly in terms of maturation from a geographically nearby (being 700 km distance away from the current cohort), healthy, 25-year-old collective that was examined using identical AI software [6]. The differences were statistically significant from late childhood onwards (boys from 8 years of age, girls from 11 years of age) and amounted to up to 0.76 years for boys and 0.74 years for girls. In their “century-long study,” Boeyer et al. described an objectively earlier onset and complete ossification of the epiphyseal joints in children born in 1995 compared to those born in 1935 [3]. Possible systemic factors are described (e.g., an increase in body mass index with changed dietary behavior) as changing socioeconomic conditions, which influence sex hormone levels in adipose tissue even before the perceptible onset of puberty. In this context, a trend towards an increase in body mass index and an earlier onset of puberty and menarche was described [21–24].

Other articles, which analyzed a current cohort using BoneXpert, also describe a trend towards a deviation of BA from CA similar to that of the current study, especially from puberty onwards, whereas no significant deviations were seen in the age group under 10 years [25]. In a Mexican population, boys aged 14–16 years and girls aged 12–14 years showed a BA about 1 year older than the CA [23].

This study has several limitations: The most important is that the retrospectively recruited collective is not a proven healthy representative collective, but one of children who had received a hand radiograph for trauma (excluding a trauma sequence). With a certain statistical probability, children with growth disorders (accelerated as well as retarded) can also be found among them. If it is assumed that the proportion of growth-retarded children is neither higher nor lower in children with trauma to the hand than in the overall population, these patients should account for only about 5% of the cohort and not significantly affect the mean, as delay and acceleration occur equally. It is also unlikely that certain ethnic groups suffer hand trauma more frequently.

In summary, the BA of the current Central European population and that of a two-decade-old population (Rotterdam cohort) differ. Whether this is due to the geographical distance between the base cities of the two cohorts or accelerated bone maturation in the last two decades must be clarified by further studies.

## Data Availability

The datasets generated and analyzed during the current study are available from the corresponding author on reasonable request.
